# Supplementary material for: Multimorbidity among Two Million Adults in China
Source: Int J Environ Res Public Health. 2020 May 13;17(10):3395. doi: 10.3390/ijerph17103395 (PMC7277827; doi:10.3390/ijerph17103395)
Supplement: Supplementary file 1 [file ijerph-17-03395-s001.pdf]

Table S1. The ICD-10 codes for the select diseases included.

NO.	Disease	ICD-10 code
1	Malignancy	C00-C13.9, C15-C25.9, C30-C34.92, C37-C38.8, C40-C41.9, C43-C45.9, C47-C54.9, C56-C57.8, C58, C58.0,C60-C63.8, C64-C67.9, C68.0-C68.8, C69-C75.8, C81-C86.6, C88-C97.9, D00.00-D00.2, D01.0-D01.3, D02.0-D02.3, D03-D06.9, D07.0-D07.2, D07.4, D07.5, D09.0, D09.2-D09.8, D10.0-D10.7, D11-D12.9, D13.0-D13.7, D14.0-D14.32, D15-D25.9, D26.0, D26.1, D27-D27.9, D28.0-D28.7, D29.0-D29.8, D30.0-D30.8, D31-D36.7, D37.01-D37.5, D38.0-D38.5, D39.1-D39.8, D40.0-D40.8, D41.0-D41.8, D42-D43.9, D44.0-D44.8,D45-D47.9, D48.0-D48.7, D49.2-D49.4, D49.6, D49.81, K31.7, K62.0, K62.1, K63.5, N60-N60.99, N84.0-N84.8, N87-N87.9, Z03.1, Z08-Z09.9, Z12-Z12.9, Z80-Z80.9, Z85-Z85.9, Z86.0-Z86.03
2	CBD	G45-G46.8, I60-I61.9, I62.0-I62.03, I63-I63.9, I64.0-I66.9, I67.0-I67.3, I67.5-I67.7, I69.0-I69.198, I69.20-I69.398, Z82.3
3	IHD	I20-I25.9, Z82.4-Z82.49
4	COPD	J40-J44.9, J47-J47.9
5	DM	E10-E10.11, E10.3-E11.1, E11.3-E12.1, E12.3-E13.11, E13.3-E14.1, E14.3-E14.9, P70.0-P70.2, R73-R73.9, Z13.1, Z83.3
6	DD	F32-F33.9, F34.1
7	CKD	E10.2-E10.29, E11.2-E11.29, E12.2, E13.2-E13.29, E14.2, I12-I13.9, N02-N08.8, N15.0, N18-N18.9, Z49-Z49.32, Z52.4, Z99.2
8	OARA	M13-M13.9, M15-M19.079; M05-M06.9, M08.0-M08.89
9	PUD	K25-K28.9, K31, K31.1-K31.6, K31.8, K31.82-K31.89
10	Cataract	H25-H26.09, H26.2-H26.9, H28-H28.2
11	HF	I50, I50.0, I50.1, I50.9
12	HT	I10, I15, I15.0, I15.1, I15.2, I15.8, I15.9, I11, I12, I13
13	Glaucoma	H36, H36.0, H40-H40.9, H42-H42.8

Abbreviations: CBD, cerebrovascular disease; IHD, ischaemic heart disease; COPD, chronic obstructive pulmonary disease; DM, diabetes mellitus; DD, depressive disorders; CKD, chronic kidney disease; OARA, osteoarthritis and rheumatoid arthritis; PUD, peptic ulcer disease; HF, heart failure; HT, hypertension.
